# Rhythm-Stratified Performance of an Artificial Intelligence–Electrocardiographic Aortic Stenosis Score: Alignment with Computed Tomography Calcium in Atrial Fibrillation

**DOI:** 10.1016/j.mcpdig.2026.100363

**Published:** 2026-04-22

**Authors:** Mustafa Suppah, Humam Abo Abdullah, Amro Badr, Vuyisile Nkomo, Reza Arsanjani, Chadi Ayoub, Dan Sorajja, David R. Holmes, Said Alsidawi

**Affiliations:** a Department of Cardiovascular Medicine, Mayo Clinic, Phoenix, AZ; b Department of Cardiovascular Medicine, Mayo Clinic, Rochester, MN

Grading aortic stenosis (AS) in atrial fibrillation (AF) is challenging because beat-to-beat flow variation lowers Doppler gradients even in severe anatomic obstruction. Contemporary work shows that patients with AF often present with low gradients despite similar or higher aortic valve calcium burden compared with patients with sinus rhythm (SR), supporting computed tomography (CT) calcium scoring as a flow-independent arbiter of severity.^1^^,^^2^ Artificial intelligence–electrocardiographic (AI-ECG) can detect moderate–severe AS with strong performance and has been proposed for screening and risk stratification.^3^ Whether an AI-ECG AS score (AI-AS) reflects hemodynamic severity (Doppler gradients) or anatomic severity by CT aortic valve calcium score (AVCS), especially in AF, remains uncertain. Although AI-ECG is trained on echocardiographic labels, the signal likely captures a composite phenotype, reflecting AS severity and downstream myocardial remodeling rather than a purely hemodynamic signal.^2^^,^^4^^,^^5^ Accordingly, in patients with established severe AS, our goal was to assess whether AI-AS is more closely associated with flow-dependent Doppler gradients or flow-independent CT-defined aortic valve calcification and whether this association varies by cardiac rhythm, rather than to validate AI-AS for diagnosing severe AS, using CT as a gold standard.

## Methods

Our cohort included adults with severe AS who underwent transcatheter aortic valve replacement (TAVR) at 3 Mayo Clinic locations (2012-2017) with available pre-TAVR 12-lead electrocardiographic, transthoracic echocardiogram, and AVCS. Our analysis included 857 patients: 544 (63%) in SR and 313 (37%) in AF groups. We defined CT-severe AVCS using sex-specific guideline thresholds (≥2000 AU for men; ≥1300 AU for women).^2^^,^^4^ Analyses included the following: (i) univariable Spearman correlations between AI-AS and mean Doppler gradient and AVCS; (ii) multivariable linear regression (dependent variable: AI-AS) using AVCS and mean gradient to evaluate whether the association with CT-based calcium score persisted after accounting for Doppler-derived hemodynamics; and (iii) receiver-operating characteristic curves for AI-AS vs CT-based severe AVCS overall and stratified by rhythm.

## Results

In the overall cohort, the AI-AS score showed consistent positive correlations with AVCS (ρ=0.14; *P*<.001) and mean gradient (ρ=0.16; *P*<.002) (Table 1). In multivariable linear regression including AVCS and mean gradient simultaneously, only AVCS remained independently associated with AI-AS (standardized β=0.15; *P*<.01) (Table 2). When stratified by rhythm, SR showed univariable correlations between AI-AS and AVCS, as well as mean gradient (ρ=0.15; *P*≤.01), but none retained independence after adjustment (AVCS: β=0.123; *P*=.10; mean gradient: β=0.024; *P*=.9) (Tables 1 and 2). In AF, correlation with Doppler mean gradient was attenuated and nonsignificant (ρ=0.10; *P*=.21), whereas the association with AVCS persisted (ρ=0.26; *P*<.001) and AVCS independently predicted AI-AS (β=0.20; *P*=.02; mean gradient: β=0.029; *P*=.73) (Tables 1 and 2). Moreover, CT-defined severe calcification (sex-specific thresholds) was present in 401 of 561 (71%) patients in SR and 213 of 297 (72%) patients in AF groups, whereas severe mean Doppler gradient criteria were met in 229 of 245 (93%) and 155 of 164 (95%) patients, respectively. For discrimination of CT-defined severe calcification, AI-AS performed modestly in the overall cohort (area under the curve [AUC], 0.585; 95% CI, 0.544-0.627; *P*<.001), was limited in the SR group (AUC, 0.560; 95% CI, 0.508-0.612; *P*=.07), and improved in the AF group (AUC, 0.635; 95% CI, 0.568-0.702; *P*<.001). In AF, AI-AS of 0.80 or greater optimized specificity (46% sensitivity and 76% specificity) and 0.745 or greater provided a more balanced trade-off (59% sensitivity and 61% specificity) (Figure).

**Table 1:** Correlation Between Artificial Intelligence Aortic Stenosis Score and Reference Measures of Aortic Stenosis Severity

Group	Variable	Spearman ρ	*P*
Whole cohort	CT calcium score	0.141	<.001
	AV mean gradient	0.159	.002
Sinus rhythm	CT calcium score	0.151	<.001
	AV mean gradient	0.188	.004
Atrial fibrillation	CT calcium score	0.256	<.001
	AV mean gradient	0.102	.21

Multivariable models: dependent variable, artificial intelligence aortic stenosis; predictors entered, CT aortic valve calcium score and AV mean gradient. Correlations are Spearman rank correlations.

AV, aortic valve; CT, computed tomography.

**Table 2:** Multivariable Linear Regression Predicting Artificial Intelligence Aortic Stenosis Score

Group	Predictor	Standardized β	*P*
Whole cohort	CT calcium score	0.151	.007
	AV mean gradient	0.185	.21
Sinus rhythm	CT calcium score	0.123	.10
	AV mean gradient	0.024	.90
Atrial fibrillation	CT calcium score	**0.20**	**.021**
	AV mean gradient	0.029	.73

Multivariable models: dependent variable, artificial intelligence aortic stenosis; predictors entered, CT aortic valve calcium score and AV mean gradient. Correlations are Spearman rank correlations. Bolded values indicate statistically significant results (*P* < .05).

AV, aortic valve.

**Figure:**
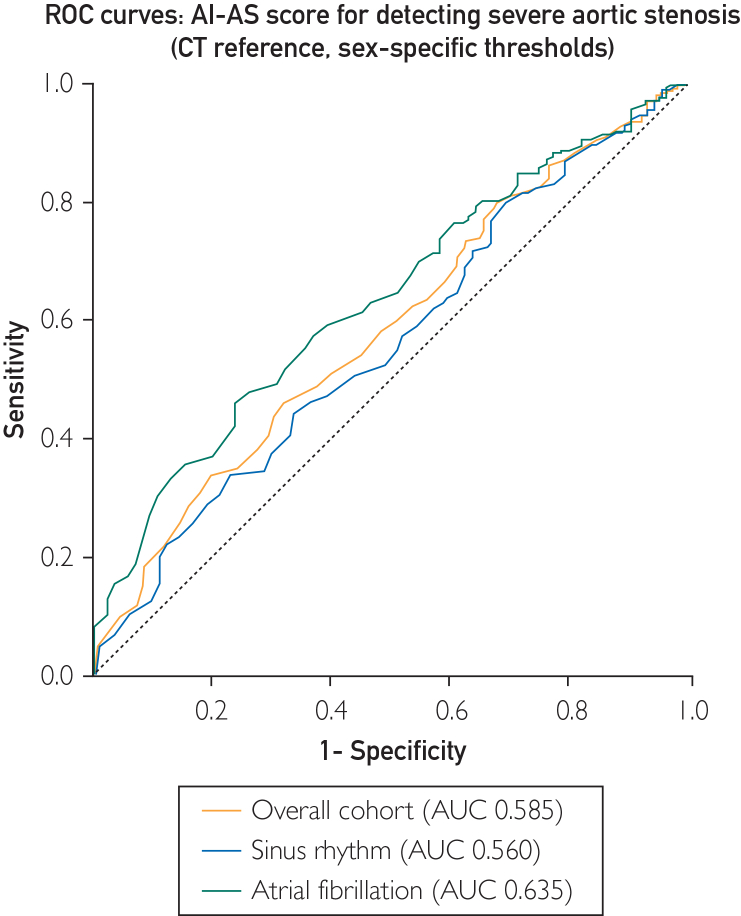
Receiver-operating characteristic (ROC) curves: aortic stenosis score for detecting severe AS (CT reference and sex-specific thresholds). Curves shown for overall cohort, sinus rhythm, and atrial fibrillation, with corresponding area under the curves (AUCs). CT severe thresholds defined as ≥1300 AU (women) and ≥2000 units (men). AI-AS, artificial intelligence aortic stenosis score; CT, computed tomography.

## Discussion

Artificial intelligence (AI) analysis of the electrocardiographic has emerged as a pragmatic way to surface patients with unrecognized AS, with prior work demonstrating credible screening performance and links to worse downstream outcomes, including post-TAVR mortality when the AI signal is positive.^3^^,^^6^ The central question is what the AI signal reflects—anatomic valve disease or flow-dependent hemodynamics by echocardiogram—and whether AF affects the signal.

When analyzed together, AI-AS score tracked with both AVCS and Doppler mean gradient, suggesting a global disease signal. When stratified by rhythm, neither AVCS nor Doppler gradient was independently associated with AI-AS in SR, whereas in AF, the associations with Doppler gradient were nonsignificant, while the relationship with CT-defined calcific burden persisted and remained independent. This rhythm-specific dissociation aligns with literature showing that AF often produces discordant, spuriously low gradients despite substantial anatomic stenosis and that CT-defined calcium score provides a flow-independent arbiter of severity in this setting.^1^^,^^2^^,^^4^

Taken together, these results refine the clinical interpretation of AI-AS. In the aggregate, the score appears to index overall disease burden, consistent with earlier work in which AI-ECG models were trained and validated against echocardiographic labels of AS.^3^ In AF, specifically, the AI-AS signal shows closer alignment with CT-defined calcium rather than with flow-dependent gradients, suggesting a potential complementary role of AS severity when hemodynamics are discrepant.^1^^,^^4^ If an AI-ECG signal flags higher mortality risk after TAVR, then pairing that signal with CT-based adjudication in AF may refine triage, identifying patients with substantial anatomic burden even when Doppler appears deceptively mild.^5^

Strengths of this study include the large real-world cohort, availability of both CT and echocardiography in the same patients, and a prespecified, rhythm-stratified analysis that mirrors clinical decision making. Limitations include the retrospective design; modest discrimination of AI-AS for CT-defined severe calcification (particularly in SR). Accordingly, the receiver-operating characteristic analyses should be interpreted as exploratory assessments of alignment with CT-defined calcific burden rather than as measures of diagnostic performance. These estimates reflect differentiation of CT-defined severe calcification within a TAVR-treated population with high-pretest probability rather than screening performance across the full spectrum of AS severity.

## Conclusion

The AI-AS score is a clinically relevant signal that increases with overall disease severity in TAVR candidates. Importantly, it shows modest correlation with CT-measured calcific burden, particularly in the setting of irregular rhythm.

## Potential Competing Interests

The authors report no competing interests.

## Ethics Statement

This study was reviewed and deemed exempt by the Mayo Clinic Institutional Review Board, which granted a waiver of informed consent owing to the retrospective design and use of deidentified data.
